# The Use of Fantasy Points to Evaluate Return-to-Play Performance After Time-Loss Injuries in the National Football League

**DOI:** 10.7759/cureus.99333

**Published:** 2025-12-15

**Authors:** Hunter S Angileri, Justin A Geier, Steven M Hadley, Daniel E McLoughlin, Madeline M Owen, Jared M May, Michael Terry, Vehniah K Tjong

**Affiliations:** 1 Orthopedic Surgery, New York University Grossman School of Medicine, New York, USA; 2 Orthopedic Surgery, Northwestern University Feinberg School of Medicine, Chicago, USA; 3 Internal Medicine, University of Pittsburgh Medical Center, Pittsburgh, USA; 4 Orthopedic Surgery, University of Washington, Seattle, USA

**Keywords:** fantasy points, injury, nfl, performance, return to play

## Abstract

Background

National Football League (NFL) “fantasy leagues,” in which fans accumulate points based on the NFL players’ in-game performance, have boomed in popularity. There is a paucity of objective measures to track player performance following injuries. Therefore, our study evaluates whether fantasy football points can serve as an assessment tool for player recovery after time-loss injuries.

Methods

In this descriptive epidemiology study, injuries in offensive skill positions over five NFL seasons (2017-2022) resulting in at least one missed game were retrospectively reviewed. Return to performance was assessed by the difference in fantasy points per game (PPG) between the injury year and the following season.

Results

Reported injuries resulted in a change of -0.50 (CI: -0.67, -0.33) PPG. Quarterbacks (QBs) had the greatest decrease in PPG at -1.95 PPG (CI: -3.47, -0.44) followed by WR at -0.33 PPG (CI: -0.53, -0.12). Injury to the shin resulted in the greatest decrease in PPG at -3.24 PPG (CI: -7.69, 1.2). Injury to the arm had the second greatest decrease in PPG at -2.65 PPG (CI: -6.78, 0.04). No association between the number of games missed and difference in PPG was found (R^2^=0.0047). Injury prior to a bye week did not alter PPG, yet injury after a bye week lead to increased loss of -1.22 PPG (CI: -1.491, -0.964). Players in the league for three years or more experienced a decline in PPG recorded of -17.10 (CI: -21.61, -12.591).

Conclusion

The use of fantasy football points may serve as an objective tool for assessing player return to performance post-injury, though correlation with rigorous clinical evaluation is still needed, especially since fantasy points lack the precision needed for injury severity and since various team-related factors may influence fantasy point performance. QBs and players with shin or arm injuries experience the greatest decline in fantasy PPG. Longer NFL careers may also contribute to performance decline, while injury timing has a lesser effect.

## Introduction

The National Football League (NFL) consistently ranks as the most watched and culturally impactful sports league in the United States. Fantasy football has likewise become a substantial facet of American sports culture. By 2022, over 50 million Americans had engaged in fantasy football, highlighting its evolution from a casual fan activity into a complex, data-intensive phenomenon that intertwines public engagement with player statistics [1]. At its core, fantasy football allows participants to build virtual teams of real NFL players, competing weekly based on individual player performances, quantified in metrics such as rushing yards, passing yards, and touchdowns. These statistics are then converted into fantasy points that determine player and team rankings [2]. This widespread fan engagement has inadvertently generated a data-rich repository of player performance that offers unique potential for correlative analysis with health trends, injury susceptibility, and recovery. Fantasy football points may therefore offer insights on player longitudinal consistency, physical durability, and statistical patterns pre- and post-injury that may complement traditional clinical metrics [1-3].

Prior literature has explored the use of fantasy football points to track professional athletes' performance post-injury, examining how these metrics may reflect recovery progress [1,2]. Bergstein et al. focused on trends in fantasy points among NFL players recovering from ankle injury, wherein fantasy points were used to track player performance before and after these injuries [2]. This study revealed that while some players were able to return to their previous levels of fantasy productivity, many displayed statistically significant decreases [2]. The study also found that fantasy points could effectively reflect the variability in recovery times across different player positions [2]. This research points to a broader potential for integrating fantasy data in clinical assessments, offering a new, publicly accessible and cost-effective way to evaluate post-injury outcomes [2]. Similarly, Kajy et al. analyzed fantasy points in evaluating players’ performance following anterior cruciate ligament (ACL) reconstruction across the NFL, National Basketball Association (NBA), National Hockey League (NHL), and Major League Baseball (MLB) [1]. The study observed that, on average, athletes showed a decline in fantasy points post-surgery, highlighting a consistent decrease in measurable performance following ACL reconstruction [1]. By analyzing fantasy football data over multiple seasons, the authors were able to quantify both immediate and long-term effects of ACL reconstruction on player productivity [1]. Given the challenges in accessing objective, long-term clinical data on professional athletes, this study showed a potential for the value of using fantasy scores as a proxy for return to performance across multiple professional sports.

Our primary objective, therefore, was to determine whether fantasy points would serve as a helpful metric of player performance following time-loss injuries across offensive positions in the NFL. The present study examined all NFL time-loss injuries, including injuries to the upper [4] and lower extremities [3,5,6] and the central axis [7,8], each of which can significantly impact offensive player performance. Several other factors beyond injury location may also be associated with player performance and fantasy point production, such as player position [9-11], timing of the injury [11-13], and tenure in the league, and were similarly examined in this study.

Taken together, there exists a complex interplay between injury location, player position, and performance. Because they serve as a metric that naturally encompasses a variety of these factors, fantasy football points may offer insights into player performance following time-loss injury previously untapped by traditional recovery metrics in sports medicine. We hypothesized that following return to play after a time loss injury, players will produce less fantasy points per game (PPG) than prior to injury across all positions and injury locations.

## Materials and methods

This investigation was a descriptive epidemiological study that retrospectively reviewed all injuries in offensive skill positions, namely, quarterback (QB), running back (RB), wide receiver (WR), and tight end (TE), across five NFL seasons from 2017-2018 through 2021-2022 that resulted in at least one game missed. Injury data such as location, games missed, week of injury, and injury reserve (IR) status were collected. Additional data on players, such as years in league, games played, and bye week timing, were also collected. The study analyzed publicly available information and did not require institutional review board approval. Injury data extraction methods followed similar methods outlined in previous studies on the epidemiology of NFL injuries [12]. Injury data were extracted from a publicly available injury report [14] and was cross-referenced with other publicly available sources [15,16]. These methods are in line with previously published protocols [12,17].

Fantasy football points are calculated from on-field statistics that are collected live by NFL personnel. Fantasy football points used in the study for each injured player were collected from publicly available online sources [14]. Since injured players participate in fewer games and thus have less on-field opportunities for scoring fantasy points, fantasy points were normalized to games played. Return to performance was assessed with the difference in fantasy PPG between the year of injury and the following season. Those with multiple injuries in the same season were treated as two separate injuries. A sub-analysis was performed by position, injury location, games missed, week of injury, and years played in the NFL.

## Results

**Figure 1:**
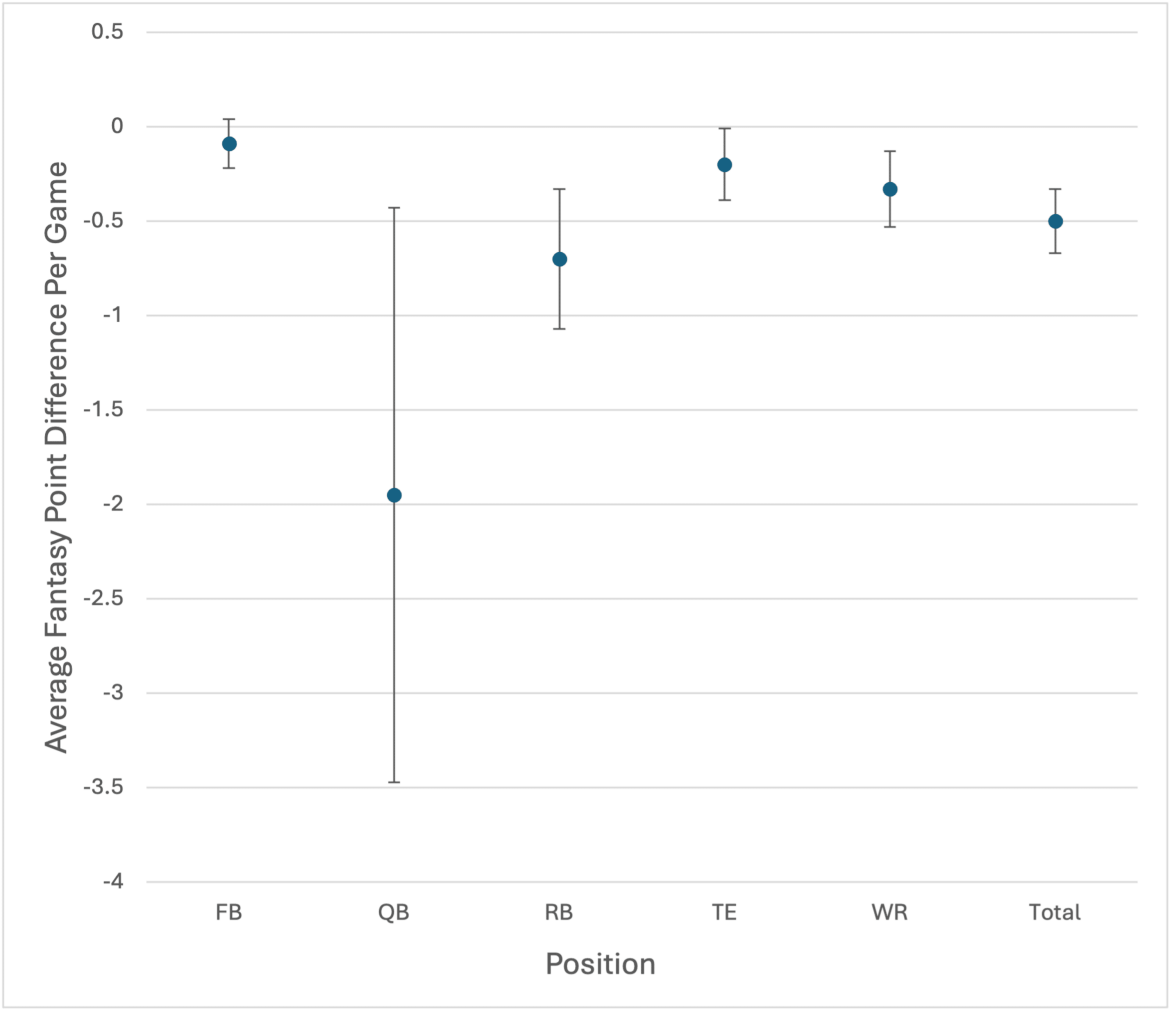
Average fantasy point difference per game by position FB=fullback, QB=quarterback, RB=running back, TE=tight end, WR=wide receiver

Across 1,216 regular season games in five NFL seasons, 2,523 time-loss musculoskeletal injuries were included in the study, with a total of 12,789 games missed (Table 1). Overall, reported injuries resulted in a decrease of -0.5 fantasy PPG played in the year after injury. By position, QB had the greatest decrease in fantasy PPG at -1.95 (CI: -3.47, -0.44) PPG followed by WR at -0.33 (CI: -0.53, -0.12) PPG (Figure 1). Among other offensive positions, RBs, WRs, and fullbacks (FBs) all demonstrated fantasy point loss after injury, with RBs exhibiting an average decrease of -0.7 (CI: -1.07, -0.34) PPG, WRs a decrease of -0.33 (CI: -0.53, -0.12) PPG, and FBs an average decrease of -0.09 (CI: -0.22, -0.04) PPG.

**Table 1:** Injuries by Position FB=fullback, QB=quarterback, RB=running back, TE=tight end, WR=wide receiver

Position	Total Injuries	Total Games Missed
FB	51	277
QB	174	940
RB	578	2,832
TE	473	2,673
WR	975	5,355
Total	2,523	12,789

**Figure 2:**
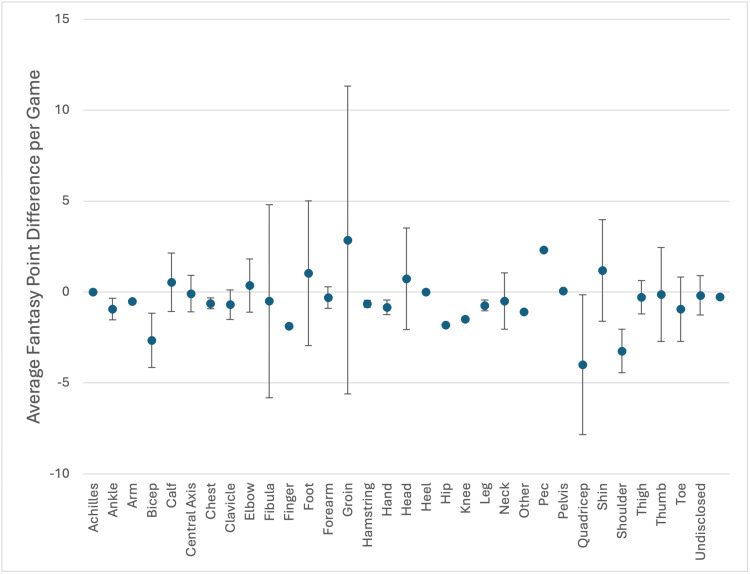
Fantasy point difference per game by injury location Injury to the shin had the greatest decrease in fantasy PPG at -3.24 (CI: -7.69, 1.2) PPG.

**Figure 3:**
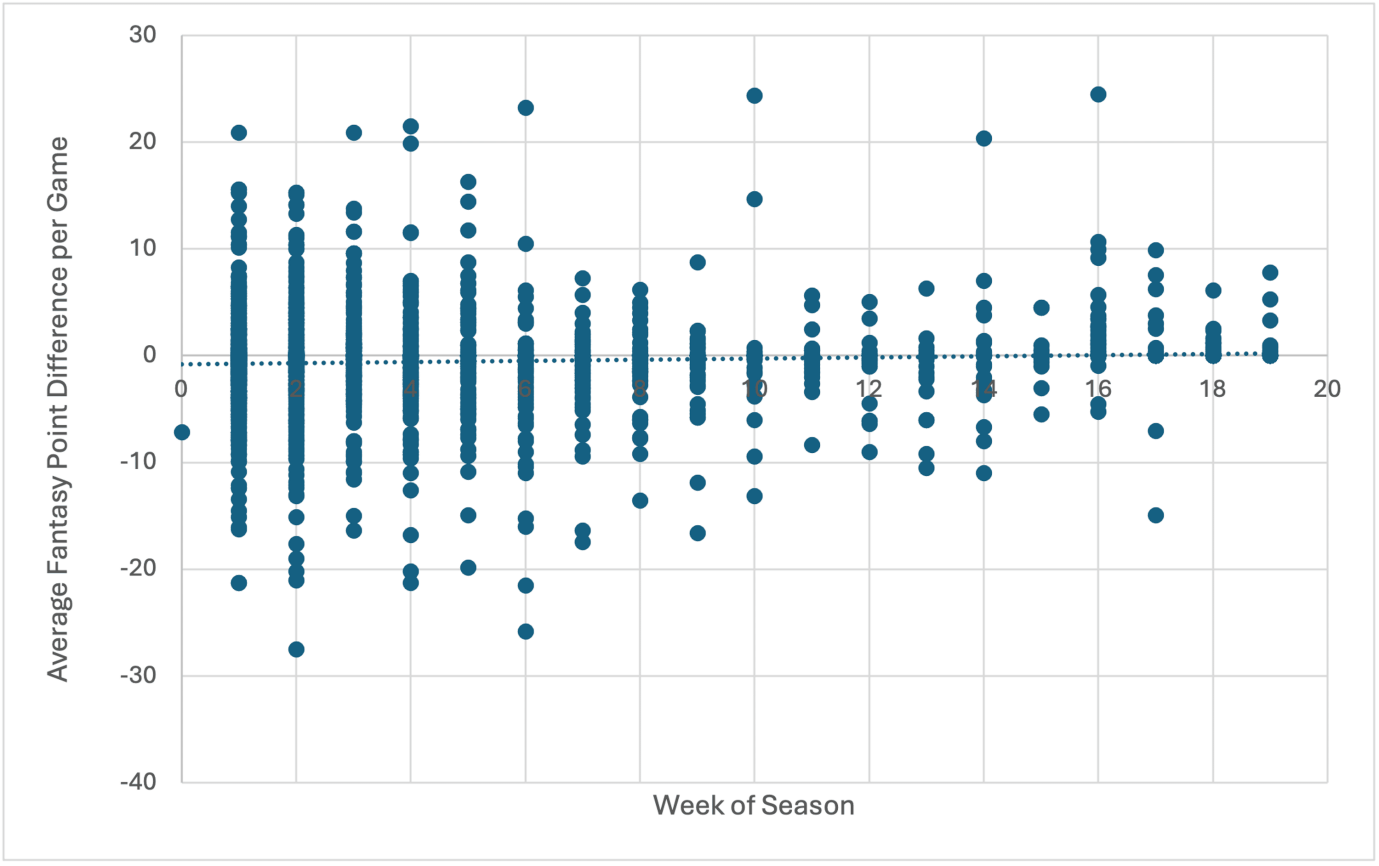
Association between games missed due to injury and fantasy point difference per game played (R2=0.0047).

By injury location (Table 2), injury to the shin had the greatest decrease in fantasy PPG at -3.24 (CI: -7.69, 1.2) PPG, followed by injury to the arm at -2.65 (CI: -6.78, 0.04) PPG (Figure 2). Data from point-earning offensive position players reveal that injuries in NFL players generally result in a decline in fantasy performance, with an average change of approximately -0.5 (CI: -0.67, -0.33) fantasy PPG. The study found no linear association between the number of games missed due to injury and difference in fantasy PPG (R2=0.0047) (Figure 3).

**Table 2:** Number of injuries by location and total games missed

Injury Location	Total Injuries	Total Games Missed
Achilles	20	159
Ankle	255	907
Arm	8	61
Bicep	2	16
Calf	49	146
Central axis	127	407
Chest	18	26
Clavicle	3	24
Elbow	9	15
Fibula	1	3
Finger	8	18
Foot	90	527
Forearm	2	8
Groin	63	233
Hamstring	267	933
Hand	19	63
Head	1	5
Heel	7	8
Hip	53	182
Knee	326	1,815
Leg	7	88
Neck	44	260
Other	1	1
Pectoralis	1	16
Pelvis	2	21
Quadricep	41	92
Shin	7	74
Shoulder	113	407
Thigh	19	43
Thumb	20	94
Toe	23	77
Undisclosed	619	5,161
Wrist	18	135

**Figure 4:**
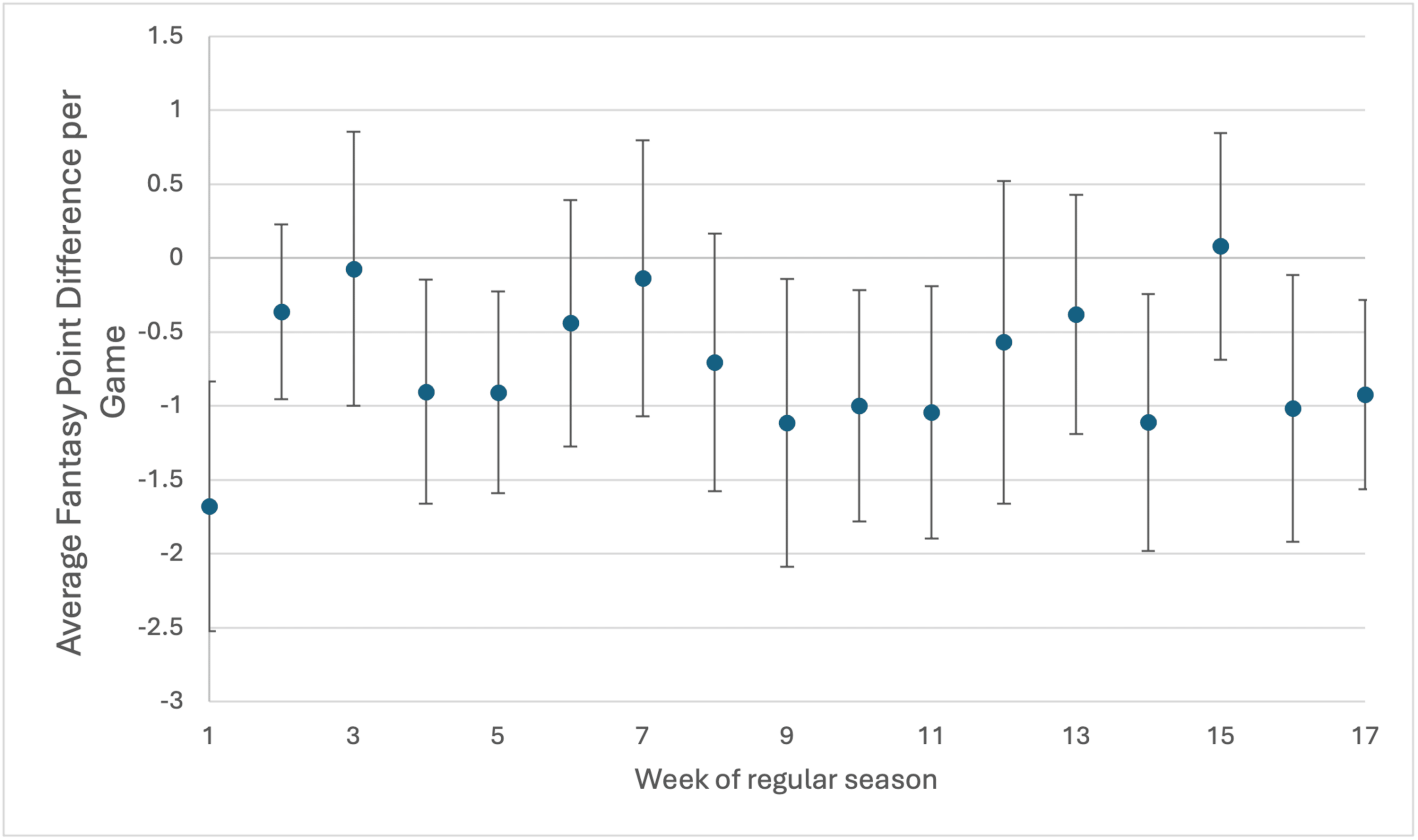
Association between the week of the regular season in which the injury occurred and fantasy point difference per game played.

The week of injury during the 17-week season also did not affect the average fantasy point difference (Figure 4). Injury before a bye week did not lead to increased fantasy point loss, yet injury after a bye week did lead to increased loss (Table 3). Finally, as the number of years played in the NFL increased, the change in fantasy football PPG also increased. On average, players with more than three years of experience in the NFL demonstrate the most severe decline in performance, with an average fantasy PPG loss of 17.10 points (95% CI: -21.61 to -12.59) compared to -3.50 PPG (95% CI: -7.64 to 0.63) for those with three or fewer years in the league (Table 3).

**Table 3:** Total games missed and change in fantasy PPG by IR status, years in league, and games played PPG=points per game; IR=injury reserve

	Total Injuries	Total Games Missed	Δ Fantasy PPG	95% CI
IR No	1,398	2,910	-0.606316425	-858 to 0.355
IR Yes	1,125	9,879	-0.368877078	-0.588 to 0.150
Years in league: ≤3	690	4,816	-3.500533618	-7.635 to 0.634
Years in league: >3	699	3,467	-17.10157368	-21.61 to 12.591
1-9 games played	647	3,838	-1.735990351	-2.045 to 1.43
10-18 games played	981	2,247	-1.700967598	-1.975 to 1.427
Injury before bye week	1,082	8,220	0.246079862	0.018-0.474
Injury after bye week	1,182	3,922	-1.22782208	-1.491 to 0.964

## Discussion

Our findings highlight the potential of fantasy football points as a novel and objective metric for evaluating player performance following time-loss musculoskeletal injuries. As the largest study to date analyzing NFL fantasy points across various injury types, our research provides valuable insights into the impact of specific injuries on player productivity. While no direct correlation was identified between games missed due to injury and changes in fantasy PPG, our results demonstrate that players experience an overall decline in fantasy PPG after sustaining a time-loss injury. While rigorous clinical evaluation for return to play is still needed, this finding underscores the potential utility of fantasy football metrics for tracking performance variations across positions, injury types, and individual players. Notably, players with shin and arm injuries exhibited more significant declines in fantasy productivity, suggesting that these injuries may present unique challenges to achieving full recovery. These findings emphasize how fantasy football point analysis may reveal injury sites and player positions that are at a higher risk of reduced performance after injury and that could benefit from more targeted rehabilitation strategies and enhanced protective measures.

Overall, the data demonstrate that injuries in NFL players generally lead to a decline in fantasy performance across all major offensive positions, with an average decrease of approximately -0.5 fantasy PPG. However, different positions vary in the degree of point loss, demonstrating that while injuries typically diminish player productivity, the magnitude and consistency of this impact may vary by position.

Notably, RBs, WRs, and FBs exhibit a uniform decrease in fantasy performance following injuries for these roles. These consistent effects likely stem from the high-contact, high-frequency demands of their positions, where physical performance is directly tied to fantasy output [10,11,18,19]. Interestingly, FBs show only a minor decline in performance, which may be attributed to their less prominent role in modern offensive schemes [10].

QBs exhibit the largest average decrease in fantasy PPG, accompanied by a notably wide confidence interval, which may reflect significant variability in the impact of injuries. This variability is likely influenced by the unique confounding factors associated with the QB position [20]. QBs rely heavily on their offensive line for protection, the availability and skill of their receivers, and the overall offensive strategy balance of passing versus running plays, all of which can fluctuate due to personnel changes [21]. Injuries may prompt teams to adjust their game plans, either to shield an injured QB or to compensate for limitations, leading to highly inconsistent performance outcomes. This variability underscores the dynamic mix of factors that make post-injury return to performance for QBs less predictable compared to other positions.

Our findings also may indicate that certain positions and injury types are linked to more challenging recoveries in terms of returning to pre-injury performance levels. RBs and WRs, who are often subjected to high-impact, rapid-movement scenarios, experience the most significant declines in fantasy productivity, particularly after injuries involving the lower extremities [2,3]. These results reinforce the value of fantasy points as a potential proxy for evaluating performance recovery, capturing how different positions and injury locations may affect functional outcomes. Given the real-time, publicly available nature of fantasy data, this metric could provide clinicians and sports analysts with a practical tool to complement traditional clinical assessments, especially for tracking long-term performance trends in athletes recovering from musculoskeletal injuries.

The significant impact of shin and arm injuries on player performance, as reflected by reduced fantasy points, likely arises from the critical functional demands these areas bear in football. These regions play essential roles in stabilization and force distribution during gameplay [5,9,22-24]. Shin injuries, including contusions, fractures, or stress-related conditions, can hinder a player’s speed, cutting ability, and lower-body coordination, all of which are vital for high-performance roles like RB and WR [5,22]. These positions demand sharp, multidirectional movements that exert intense stress on the shin and lower leg, making even minor injuries particularly harmful to agility and balance [25]. Furthermore, the high-impact nature of turf surfaces commonly used in NFL stadiums amplifies ground reaction forces, increasing the likelihood of recurrent injuries upon a player’s return [6]. Research indicates that optimal shin injury prevention involves intentional stress reduction [25]. Together with the findings of this study, this may suggest that performance could be optimized through effective load management strategies if a decrease in fantasy production is observed.

Arm injuries, including fractures, ligament tears, and dislocations, can be particularly disruptive, especially for positions that rely heavily on arm function, such as QBs and WRs [18,26,27]. The arm is crucial for tasks such as catching, ball handling, and providing protection during tackles. Even after clinical recovery, players may experience residual stiffness, reduced grip strength, and persistent pain, all of which impair the fine motor skills necessary for accurate passes and secure catches. Research indicates that upper-extremity injuries often lead to long-term functional deficits that, while sometimes subtle, can significantly hinder gameplay and prevent players from regaining their pre-injury performance levels [4,20,27]. Additionally, these injuries can result in substantial time away from play. A 10-year review of the NFL injury monitoring system revealed that hand injuries accounted for an average of 30-40 days lost, while elbow and forearm injuries led to an average of 22 days lost from competition [4,26]. Some of the deleterious effects of shin and arm injury may be mitigated if careful tracking of fantasy production allows for increased awareness of deficits in player performance and factors into coaching and training decision made for an injured player.

Trends in fantasy point loss post-injury may reveal significant differences based on a player’s tenure in the league and the timing of the injury relative to the bye week. Players with more than three years of NFL experience exhibit the most substantial decline in performance compared to those with three or fewer years in the league. This disparity may be attributed to the cumulative physical wear and tear associated with longer careers, as well as the financial and career security tied to the three-year pension threshold, which might compel veteran players to continue playing through injuries at a reduced capacity. Furthermore, age-related performance declines and the accumulation of prior injuries likely intensify the performance impact for players with longer tenures [28]. These effects persist past the player’s time in the league, as prior studies have reported that longer NFL careers are associated with higher rates of whole-person impairment, an objective measure of disability [28,29]. As such, tracking fantasy production may serve as a tool for players to track and manage career decisions involving recovery and retirement goals.

Fantasy football point production by the timing of injuries in relation to a team’s bye week also may prove a useful insight, as it may highlight the role of scheduled recovery periods in performance outcomes. Players injured before their bye week showed a slight improvement in fantasy performance, with an average increase of 0.25 PPG. This improvement likely reflects the additional recovery time afforded by the bye week. Conversely, injuries occurring after the bye week were associated with a significant decline in performance, with an average loss of 1.23 PPG. While prior research found no link between bye week timing and increased injury rates [12], bye week timing may influence return-to-performance outcomes for players suffering time-loss injuries. Recovery periods scheduled later in the season may leave insufficient time for full rehabilitation, resulting in persistent performance deficits [30]. This finding aligns with previous research on professional hockey players, which identified a positive association between consecutive games played and injury rates [13]. Interestingly, whether a player was placed on the IR list - a designation typically reserved for more severe injuries - did not correlate with exacerbated PPG losses.

Collectively, these findings may emphasize the nuanced impact of injuries on performance, influenced by career stage and recovery timing, using fantasy football points as a proxy for return-to-performance outcomes. They also may highlight the potential of fantasy football points as a novel tool to provide insights into player recovery that extend beyond the physical manifestations typically assessed through medical and training evaluations.

This study does have some limitations. As with any descriptive epidemiological study, this study relies on a retrospective analysis of data collected prior to the conception of the present study; therefore, the data exists in the detail and format in which it was originally recorded and was not monitored for consistency at the time of collection. To address this potential limitation, multiple distinct databases with independent means of data collection were cross-referenced to ensure accuracy of the data used in this study.

One notable limitation of this study was the inability to draw a meaningful connection between difference in fantasy football points and injury severity because the number of games missed was not usable as an appropriate proxy for injury severity. The publicly available data sources also did not have more specific information (such as sprain vs. fracture) to characterize the injury severity further. Because we found no correlation between the number of games missed and fantasy PPG difference after return to play, our analysis also reveals that fantasy points may have limited utility in evaluating broader injury epidemiology within the NFL. Although fantasy points provide granular, game-based data on player output, they do not appear to directly reflect the severity of injuries, limiting their capacity as a comprehensive tool for injury epidemiology. In addition, other factors, such as coaching changes, offensive scheme changes, quality of opposition, weather, and psychological factors in recovery, may influence PPG. These findings indicate that while fantasy points can capture recovery outcomes on an individual level generalized across injury types, they lack the precision needed for severities within a population-based framework. Therefore, there remains a critical need for more objective, standardized measures to evaluate in-game performance and recovery post-injury, especially for establishing reliable epidemiological data on injury patterns in the NFL.

The heterogeneity of factors influencing a player’s return-to-sport timeline and performance further limits the use of fantasy points for evaluating outcomes following less common injuries or those that did not result in missed playing time. However, the large sample size of this study - encompassing players across all offensive positions and injury types - provides sufficient power to analyze the impact of time-loss injuries on fantasy football performance within broader categories, such as injury location or player position. Future studies could address these limitations by adopting a prospective design to track players before, during, and after injuries, incorporating traditional sports medicine evaluations alongside fantasy production metrics. Such an approach would allow for more standardized, objective measures of in-game performance and recovery, improving the reliability of epidemiological insights. Moreover, due to the inherent, aforementioned limitations with using fantasy points, statistical analysis was not able to account for these confounding variables, especially since observations related to severity, prediction, and long-term decline are not independent of these confounding variables.

In summary, while fantasy points alone are insufficient for detailed epidemiological analyses, they hold significant promise as a low-cost, scalable method for tracking individual recovery outcomes in professional football. Future research should explore integrating fantasy points with clinical metrics to better understand recovery trajectories, refine rehabilitative protocols, and improve injury prevention strategies. By further investigating these applications, we can develop a more robust framework for assessing athletic performance post-injury, benefiting both medical research and sports performance analytics.

## Conclusions

The use of fantasy football points may be a useful objective measure to track an individual professional football player's return to play performance after a time-loss musculoskeletal injury, though correlation with rigorous clinical evaluation is still needed. There are unique positions and injuries that have a worse return to performance as measured by the difference in fantasy PPG between the year of injury and the following season.
